# Hybrid Laser–Etching Fabrication of High-Density Antireflective Microstructures on DLC-Coated Silicon Using Burst-Mode Bessel Beam Processing

**DOI:** 10.3390/nano16181131

**Published:** 2026-09-10

**Authors:** Xxx Sedao, Javier Prada-Rodrigo, Agathe Cavanna, Ionut-Alexandru Bunea, Antoine Gonon, William Ravisy, Yannick Bleu, Christian Seassal, Yves Jourlin, Laurent Dubost, Nicolas Crespo-Monteiro, Razvan Stoian, Yoan Di Maio, Claire Deeb, Isabelle Verrier, Emilie Gamet

**Affiliations:** 1 Laboratoire Hubert Curien, Unité Mixte de Recherche—Centre National de la Recherche Scientifique 5516 (UMR CNRS), Université Jean Monnet, Université de Lyon, 42000 Saint-Etienne, France; 2 Grupo PEASER, Instituto de Óptica (CSIC), C/Serrano, 121, 28006 Madrid, Spain; 3 Centre National de la Recherche Scientifique (CNRS), Ecole Centrale Lyon (ECL), Institut National des Sciences Appliquées (INSA) Lyon, Université Claude Bernard Lyon 1, École Supérieure de Chimie Physique Électronique de Lyon (CPE) Lyon, Institut des Nanotechnologies de Lyon (INL), Unité Mixte de Recherche (UMR) 5270, 69134 Ecully, France; 4 National Institute of Laser, Plasma and Radiation Physics, Str. Atomistilor, 409, 077125 Magurele, Romania; 5 Groupement d’Intérêt Économique (GIE) Manutech-USD, 20 Rue Prof Benoit Lauras, 42000 Saint-Etienne, France; 6 Institut de Recherches en Ingénierie des matériaux de Surface, IREIS, HEF Groupe, Av. Benoît Fourneyron, 42160 Andrézieux-Bouthéon, France; 7 Thales Research and Technology, Campus Polytechnique, 1 Avenue Augustin Fresnel, 91767 Palaiseau, France

**Keywords:** femtosecond laser, laser-induced etching, diamond-like carbon, silicon, selective etching

## Abstract

We demonstrate a novel method for fabricating densely packed, high-aspect-ratio cavities (depth to diameter ratio > 1) in silicon wafers coated with diamond-like carbon (DLC). The process combines femtosecond laser irradiation with subsequent wet etch (potassium hydroxide-based) or plasma etch (chlorine- and oxygen-based). The femtosecond laser is used to pattern precise openings in the DLC layer, exposing the underlying silicon, which is then rapidly etched to form well-defined cavities. This combined laser ablation and etching approach enables high-throughput fabrication of densely packed micrometer cavities and offers scalability toward industrial production. The resulting hybrid DLC-coated silicon structure, featuring densely packed cavities, may exhibit antireflective properties in the far-infrared spectrum range.

## 1. Introduction

Laser pulses at femtosecond timescales have been extensively employed in material processing, especially for precise materials structuring at the micro- and nano-scales that enables surface functionalization towards the control of optical, mechanical, or chemical surface properties in research as well as industrial-scale applications. Over the past decades, femtosecond laser ablation has been implemented for a wide range of applications across fields such as medicine, microfluidics, micro-optics, and MEMS [1,2,3,4]. Owing to their extremely high peak intensity and ultrashort pulse duration, femtosecond lasers enable high-precision machining while minimizing the heat-affected zone, making them particularly attractive for processing hard coating materials such as titanium nitride (TiN), zirconium nitride (ZrN), and diamond-like carbon (DLC).

DLC, a form of amorphous carbon with a significant fraction of sp^3^ bonds, has attracted considerable attention in both scientific research and industrial applications [5]. DLC coatings combine high hardness, low friction coefficient, excellent chemical stability, and optical transparency, making them suitable for a wide range of applications, including photolithography resist materials and protective hard coatings [6]. In particular, DLC-coated silicon substrates are of interest for infrared optical systems, where the coating provides excellent mechanical protection against scratches and erosion caused by hard airborne particles such as sand and dust while maintaining favorable optical properties [7]. Despite these advantages, silicon exhibits a high refractive index, resulting in significant Fresnel reflection losses at the air–substrate interface that reduce infrared transmission. One effective strategy for mitigating these losses is the fabrication of subwavelength surface structures that create a gradual effective refractive-index transition between air and the substrate, thereby reducing surface reflectivity [8,9]. In the present work, this structuring approach is implemented on DLC-coated silicon, where the DLC layer serves multiple functions: it acts as a protective coating during processing and as a functional layer required for the subsequent etching process. For the targeted infrared application, the surface structures must be arranged in a periodic array with a pitch of 2 µm. Among the available laser processing strategies, a Bessel–Gauss beam is particularly well suited for fabricating periodic structures at this spatial scale [10]. Although more often the Bessel beam is used for texturing or patterning transparent materials [11,12,13], recent studies suggest that the Bessel beam in the near-infrared spectrum is also suitable for opaque material drilling, such as metal and semiconductors [6,14], albeit multiple laser pulse irradiation is necessary [15,16]. Their enhanced drilling performance is attributed to the self-reconstruction property of the Bessel beam, which enables the main lobe to regenerate and propagate through the drilling channel even in the presence of nanoscale scatterers.

For the above-mentioned application in an infrared optic system, a large-area surface has to be laser patterned, typically 2-inch wafers. Bessel-beam processing is commonly performed using a stationary laser beam while the sample is translated beneath it. For the multiple-pulse irradiation as discussed previously, the process would be set in a move-and-shoot strategy. However, industrial-scale manufacturing requires substantially higher throughput, motivating the development of on-the-fly processing strategies, in which the wafer remains in continuous motion throughout fabrication. In this context, GHz burst-mode femtosecond laser irradiation offers significant advantages. A GHz burst consists of a sequence of closely spaced femtosecond pulses separated by sub-nanosecond to nanosecond intervals, enabling multiple pulse–material interactions at nearly the same surface location during continuous sample translation.

In this work, we present a hybrid fabrication approach that combines an axicon-generated femtosecond Bessel beam operated in GHz burst mode with a subsequent chemical etching step to fabricate high-aspect-ratio periodic structures (ratio of depth-to-diameter > 1) on one side of a DLC-coated silicon substrate. The novelty of the present work does not lie in the individual use of these techniques, but rather in their integration into a single fabrication strategy for the rapid and scalable production of microstructures on silicon. Specifically, the DLC-coated silicon substrate is used as a laser-patternable hard-mask system, followed by parallel silicon removal through chemical or plasma etching. The proposed approach enables the direct fabrication of dense arrays of localized openings in the DLC layer while minimizing the influence of the sample translation during each burst. The subsequent etching step then removes silicon simultaneously beneath all laser-patterned openings, providing a potentially scalable route compared with direct point-by-point laser machining of the complete cavity depth. The proposed method enables the fabrication of densely packed cavity arrays in an on-the-fly configuration while maintaining high processing speed and scalability, demonstrating its potential for industrial implementation.

## 2. Materials and Methods

The main element of the experimental setup was the femtosecond laser system with GHz burst mode capability. The laser system (Pharos, Light Conversion, Vilnius, Lithuania) delivered femtosecond laser pulses with a pulse duration of 220 fs at a near-infrared wavelength of 1030 nm. Thanks to the pulse-picking capabilities of the system, the laser pulses were provided at tunable repetition rates up to 1 MHz, reaching a maximum output power of 10 W. As described above, the GHz mode combines up to 25 pulses at a GHz repetition rate inside a packet (the burst), and the repetition rate of the bursts is at a kHz rate. For this, we use ppb (pulses per burst, or intra-pulse number) to designate the number of pulses in a GHz burst.

**Figure 1:**
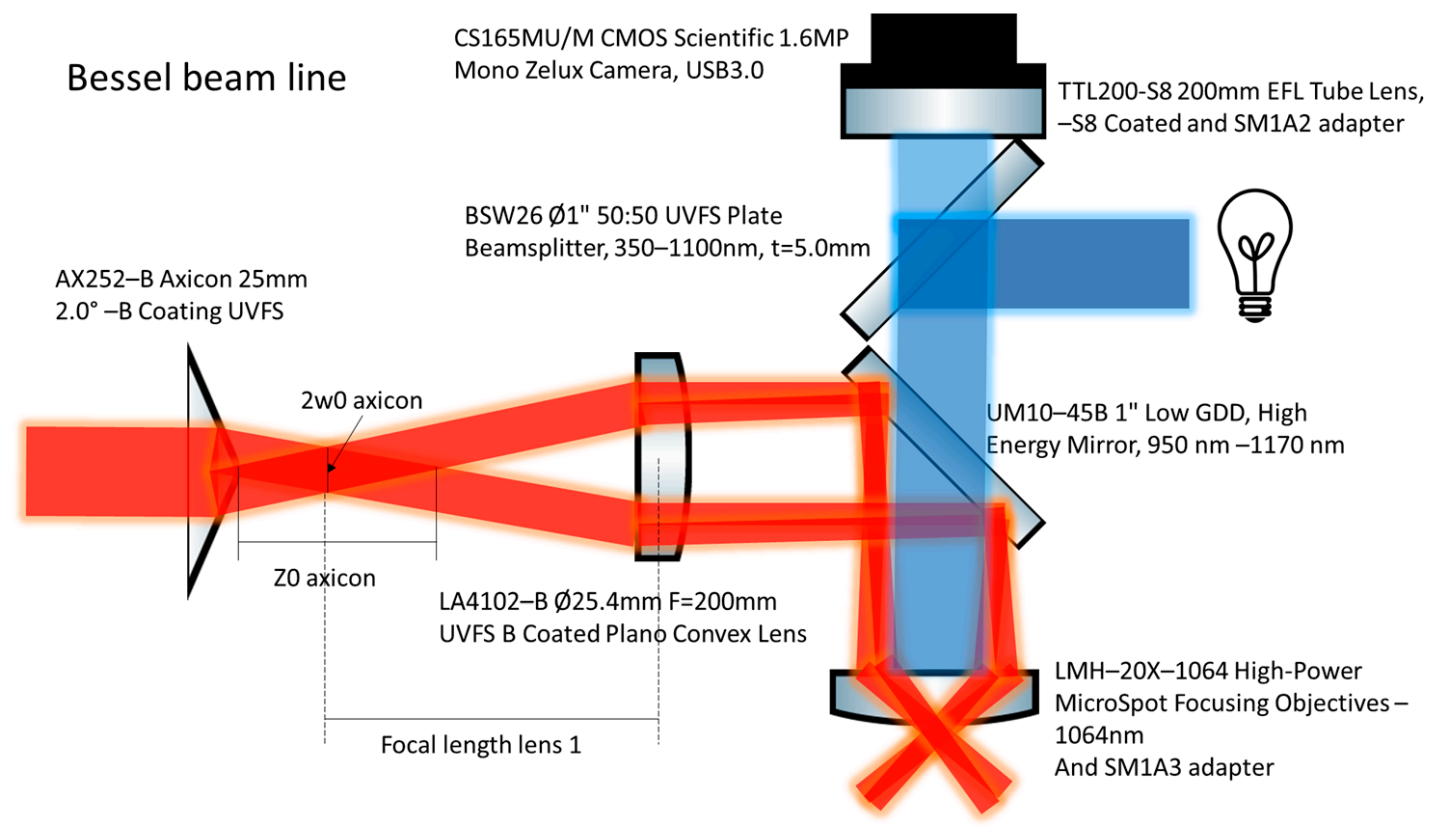
Experiment setup with Bessel beam for material processing and in situ vision system used for visualizing sample surface during process.

A high-quality axicon or conical lens is chosen as an efficient way for generating the so-called Bessel–Gaussian beam [10]. When collimated Gaussian laser beams pass through the axicon, the conical intersection of wavefronts upon propagating through the axicon creates an interference pattern which is characterized as the zeroth-order Bessel beam. Then, a 4f telescopic imaging system was used to project the beam on the sample surface, with a microscope objective as the final element (Figure 1). All optic elements were purchased from Thorlabs. As shown in the schematic in Figure 1, the apex angle of the axicon is 2°. The 4f system consists of a 200 mm plano-convex lens and a 20× objective. A visualization system is also placed in conjugation with the focusing objective through a 200 mm tube lens for in situ vision inspection. The Bessel beam waist diameter (the central core, FWHM) is 2ω_0_ = 1.15 µm (dedicated calculations can be found in the Supplementary Section S5). In the following discussions, pulse energy is used as the laser processing parameter. The pulse energy is the total energy maintained in the laser pulse. The contrast between the first side-lobe and the central peak of the Bessel beam intensity was estimated between 16% and 25%.

Single-side polished silicon wafers of type n, 1620 Ohm.cm, were purchased from Neyco (Neyco, HEF groupes, Andrézieux-Bouthéon, France). The thickness of the wafers were 400 µm, and the surface finishing of the polished side had an Ra of less than 0.5 nm. The crystal orientation 110 was chosen for the desired etching process [17,18,19]. The wafers then underwent a thin film deposition process for the DLC coating. The DLC coating consists of an amorphous hydrogenated carbon layer deposited by plasma-enhanced chemical vapor deposition (PECVD) in a HEF TSD-550 industrial chamber. The chamber was equipped with a rotating vertical barrel substrate holder of diameter 550 mm and multiple electron cyclotron resonance microwave plasma sources along the height. The microwave was first started in an argon atmosphere for plasma cleaning of the substrates before treatment, then acetylene (C_2_H_2_, CAS 74-86-2, 99.99% Messer) was introduced at a constant flow rate of 135 sccm (standard cubic centimeters per minute) for the coating. A voltage bias was applied to the substrate holder and samples to increase ion bombardment, allowing proper plasma cleaning and fine-tuning of the deposited DLC mechanical and optical properties [20,21].

The DLC-coated silicon samples were then placed on motorized XYZ translation stages from Aerotech (ANT130 series, Pittsburgh, PA, USA). The control of the number of pulses and laser fluence was realized via the laser system software. The irradiation was carried out in air, at ambient conditions of temperature and pressure.

Wet anisotropic etching of DLC-coated silicon was primarily carried out using alkaline solutions such as potassium hydroxide (KOH). We etched the specimen in 35 wt.% KOH water solution at 75 °C. The etch time and solution concentration were optimized prior to the fabrication of the structures investigated in this report.

Plasma etching was carried out at the Institute of Nanotechnology of Lyon (INL). The samples were placed in an ICP-RIE (Sentech SI 500) plasma etch system at 0.7 Pa and 20 °C, with a gas mixture of Cl_2_ (22 sccm) and O_2_ (3 sccm). The chamber was first passivated, followed by an etching step of 800 s.

The surface morphology of the laser impacts was analyzed using a JEOL IT-800 SHL scanning electron microscope (SEM) (JEOL Ltd., Tokyo, Japan) equipped with a field emission gun, providing high-resolution imaging of the generated structures. The SEM imaging was conducted in secondary electron mode under high vacuum with an acceleration voltage of 5 to 10 kV. SEM images were captured at various magnifications in order to examine the patterns formed at different scales. For certain cases, a Focused Ion Beam (FIB) of an FEI Helios Nanolab 600i Dual-beam workstation (FEI, Lausanne, Switzerland) was used to reveal the 3-dimensional profiles of the laser-structured surface. In most cases, the depth of cavities fabricated by laser ablation was estimated by tilting the samples at 35° in the SEM chamber. This configuration enables a perspective view of the cavities, facilitating their morphological/topographical inspection.

Numerical simulations of the optical response were performed using MC Grating software [22], based on the Fourier modal method, also known as rigorous coupled-wave analysis (RCWA) [23]. The investigated surfaces were modeled as corrugated structures under plane-wave illumination. The geometrical parameters were set according to the dimensions targeted by the laser/etching process and to the spectral range of interest. The nominal period and cavity diameter were Λ = 2 µm and Ø = 1.6 µm, respectively. The calculated reflectance and transmittance spectra were then used to assess the optical response of the structured surfaces and to compare it with the corresponding flat references.

## 3. Results

In this section, the results are presented as follows. First, the laser processing conditions are investigated in a classical single-shot configuration, with the aim of identifying the process window, in particular the laser pulse energy and the number of pulses required to achieve the targeted structural units and surface matrix. Second, the laser process is transitioned from single-shot operation to burst mode with GHz intra-burst repetition rates. This step is essential to ensure scalability of the process for future industrial production. Third, the post-laser etching processes are discussed, as they are also critical for achieving the designed surface structures. The optical simulation is followed to highlight the potential anti-reflection property of the surface in the infrared spectrum range.

### 3.1. Bessel Beam Ablation in Single-Shots Mode (Without the Burst)

**Figure 2:**
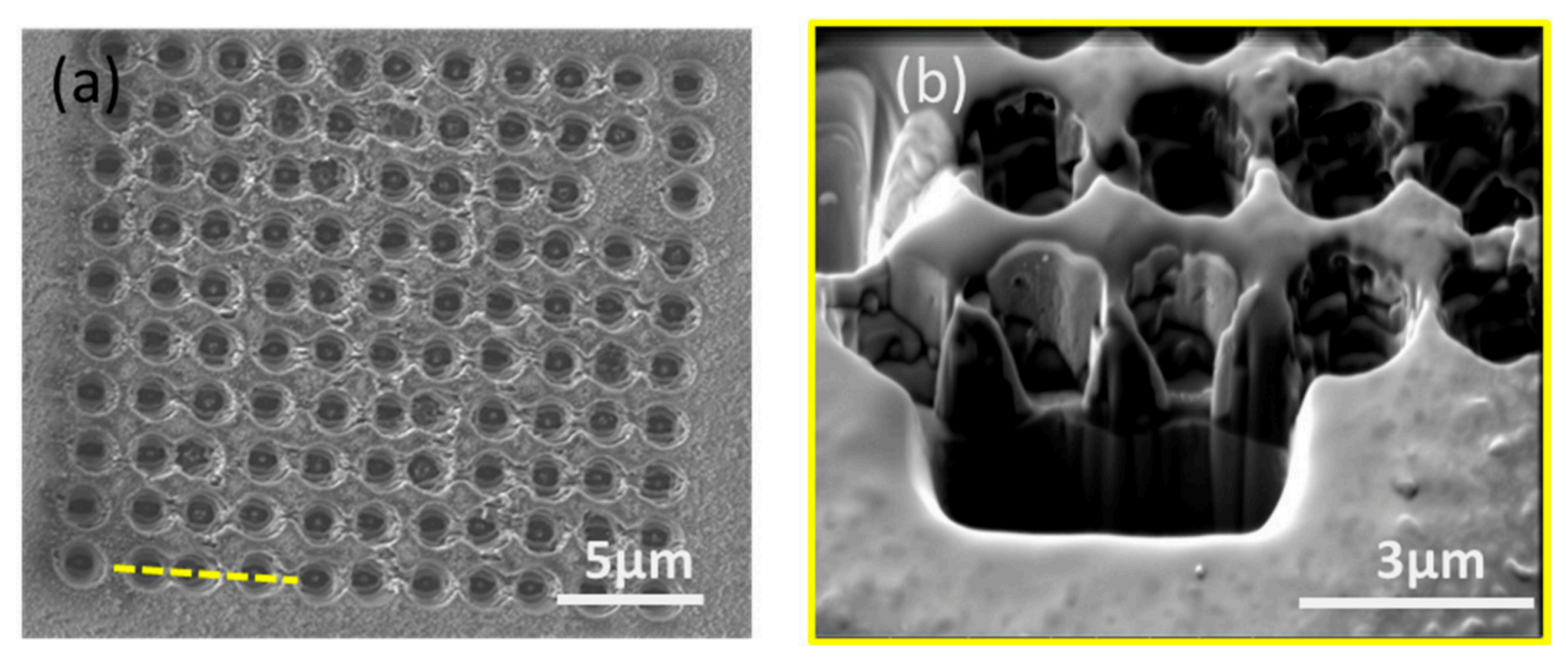
SEM images illustrating laser cavity patterning. An 11 × 10 cavity matrix was made onto DLC on Si substrates with 20 pulses at 1 µJ/pulse for each cavity; (**a**) top view of the entire matrix. The yellow dotted line indicates the site for the cross-section inspection; (**b**) FIB milled cross-section of 2 cavities from the matrix, highlighting the 3D profile of the cavities.

Prior to the Bessel beam test, a preliminary study was carried out to investigate the threshold and DLC response to the femtosecond laser irradiation (details of this investigation can be found in the Supplementary Section S1). It was identified that the single-shot ablation threshold is approximately 1.6 J/cm^2^ for a Gaussian beam configuration and peak fluence (in agreement with literature [24,25]), and that Low Spatial Frequency Laser-induced periodic structures (LSFL) are formed perpendicular to the laser polarization, similar to metallic or semiconductor materials [26]. These observations suggest that the DLC film behaves similarly to a metallic surface under intense femtosecond laser irradiation. The Bessel beam was chosen due to its capacity to increase the depth of the laser-induced crater and reduce the diameter of the crater compared to a Gaussian beam [16]. The Bessel beam generated in the experiment contains a core surrounded by concentric higher-order rings, with most of the energy concentrated at the core of the Bessel beam. The energy of the Bessel beam is accumulated along the axis of the propagation direction. The calculated Bessel beam values of the core along the diameter and axis of the propagation direction were 1.6 µm and 400 μm, respectively. The classic approach of “move-and-shoot” configuration was applied. In this configuration, the cavities were fabricated one after another. Each cavity received 20 pulses, and pulse energy was fixed at 1 µJ/pulse. An example of such a cavity matrix is shown in Figure 2, where Figure 2a presents an SEM micrograph overviewing an 11 × 10 matrix with 2 µm pitch. The cavities are approximately 1.6 µm in diameter, hence matching the aforementioned numerical design. In order to access the depth profile of the cavities, FIB cross-section means were applied. The yellow dotted line in Figure 2a indicates the selected site for FIB cross-section inspection. Figure 2b is an FIB-prepared cross-section SEM image revealing the 3D profile of 2 cavities from the matrix that was used to prepare certain cross-section samples for the inspection of the depth profile of laser-fabricated cavities. The depth of the cavities is measured from the cross-section to be approximately 2 µm. The depth-to-diameter aspect ratio is greater than 1 for each individual cavity.

Notwithstanding a successful demonstration of the laser process for fabricating the cavities with the desired geometry, the “move-and-shoot” configuration is a time-consuming process configuration: the time consumption is mainly due to the sample holding mechanism having to stop for laser irradiation and then displace the sample to the next position of laser illumination. The repetitive acceleration and deceleration limit the fabrication process from running in an industrial setting. For instance, if we estimate (based on Aerotech ANT130 linear translation stages specifications (Aerotech, Inc., Pittsburgh, PA, USA)) the travel time for a motorized linear stage at approximately 50 milliseconds (ms) for a 2 µm distance “move”, and 2 ms for 20 laser pulses irradiation “shoot”, a typical ‘move-and-shoot” cycle takes 52 ms. Additionally, the average travel speed (including the stage ramping, constant moving, and shoot time) is therefore 0.038 mm/s. As a consequence, for processing a 2-inch wafer, it would take 9028 h, or more than 53 weeks (one year) of non-stop laser processing to accomplish, which makes this process literally inapplicable for manufacturing. Therefore, in the following, we explore possible alternative solutions that would allow the process time to be acceptable from an upscaling point of view. The most promising strategy is an “on-the-fly” type of laser processing scheme, during which the sample and the translation stages keep moving continuously without a stop. If we take a moderate stage translation speed of 25 mm/s, the 2-inch wafer can be fully laser patterned in less than 14 h. The challenge is then to remove the necessary amount of material without stopping to achieve the desired geometry. The burst mode of laser pulses should provide a clear advantage for the delivery of the desired laser photon energy in “on-the-fly” mode, as explained in the following section.

### 3.2. Bessel Beam Ablation in Burst Mode

**Figure 3:**
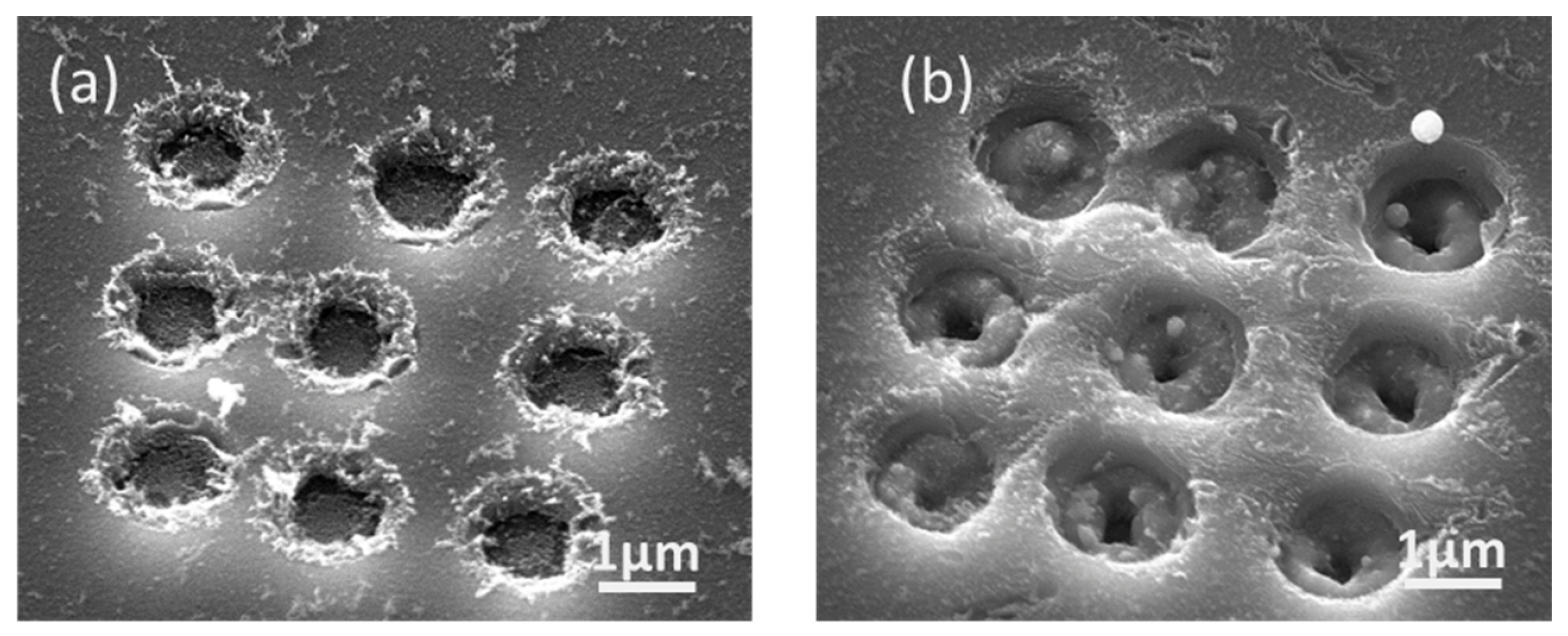
SEM images (samples tilted at 35° in the SEM chamber) illustrating laser cavity patterning; 3 × 3 cavity matrices were made onto DLC on Si substrates with varying laser conditions: (**a**) 1 burst of 25 ppb; and (**b**) 5 bursts of 25 ppb.

Compared with classic single-pulse irradiation at kHz repetition rates, burst-mode processing can enhance ablation efficiency because subsequent pulses interact with material that has already been excited or modified by preceding pulses within the same burst [27]. Burst mode with MHz and GHz repetition rates is believed to be beneficial for enhancing ablation efficiency and obtaining greater depth-to-diameter aspect ratio structures in semiconductors [27,28]. We also evaluated the ablation depth of the DLC layer when operating in classic kHz mode and in burst mode. The depth difference at different repetition modes and the advantage of applying burst mode can be found in the Supplementary Section S1. In this study, we maintained our laser process configuration at GHz burst regime only (this choice was made with the consideration of future upscaling—the upscaling is more likely to be made with another laser with burst option from Amplitude SAS (Talence, France), with runs at GHz bursts configuration only). We kept the burst energy approximately equivalent to the pulse energy in the previous section (1 µJ/pulse in simple pulse repetition mode). A burst configuration of 25 pulses per burst (ppb) is chosen; each individual ppb carries 40 nJ of energy, hence 1 µJ/burst (when higher energy per burst was chosen, cracks formed between individual cavities in the matrix). This configuration allows for ablation removal of 80–100 nm per burst, roughly the same order of magnitude of the material removal quantity as the classic single shots case reported in Section 3.1. Samples of 1 burst and 5 bursts were selected to prepare 3 × 3 cavity matrices at 2 µm pitch for SEM observation. A few typical observation micrographs are illustrated in Figure 3. The SEM micrographs are taken with a 35-degree tilt angle: Figure 3a for 1 burst irradiation and Figure 3b for 5 bursts. The depth estimation of the ablation with the Bessel beam irradiation in burst mode was made during SEM observation. The DLC removal with 1 burst of 25 ppb is about 4 nm/ppb, and the removal rate with 5 bursts of 25 ppb drops slightly, reaching 3.2 nm/ppb. It is worthwhile to note that the irregularity of the cavity’s spacing shown in Figure 3 was due to the precision of the sample positioning of the translation stages; when operating in on-the-fly mode, the positioning precision would be significantly improved (more detailed discussion on positioning precision is found in Supplementary Section S4. The more bursts, the deeper the cavities get. The cavities in both cases are clearly defined and crack-free at the edges.

### 3.3. Wet Etch in KOH Solution

Chemical etching of Si in KOH solution comprises two main steps: the OH−catalyzed conversion of Si–H surface bonds to give Si–OH (and H_2_) and the breaking of the polarized Si–Si back-bond by reaction with water. The following formulas are used to describe the reaction path [29]:

Si + 2OH^−^ → Si(OH)_2_^2+^ + 4e^−^ (1)

4e^−^ + 4H_2_O → 4OH^−^ + 2H_2_ (2)

Si(OH)_2_^2+^ + 4OH^−^ → SiO_2_(OH)_2_^2−^ + 2H_2_ (3)

The reaction resultants are either gaseous product (H_2_) or soluble products SiO_2_(OH)_2_^2−^; hence, solid silicon is removed through this reaction chain. In addition, the atomic arrangement depends on the surface: (100), (110), and (111) surface atoms have 2 and 3 Si–Si back-bonds, respectively [30]. Therefore, for the (111) case, the surface atoms are more firmly anchored in the lattice. It is more difficult to form the transition-state intermediate and the surface is more stable (see Supplementary Section S2 for information). The Si (110) was chosen as substrate for DLC coating for laser patterning and subsequent wet etching.

**Figure 4:**
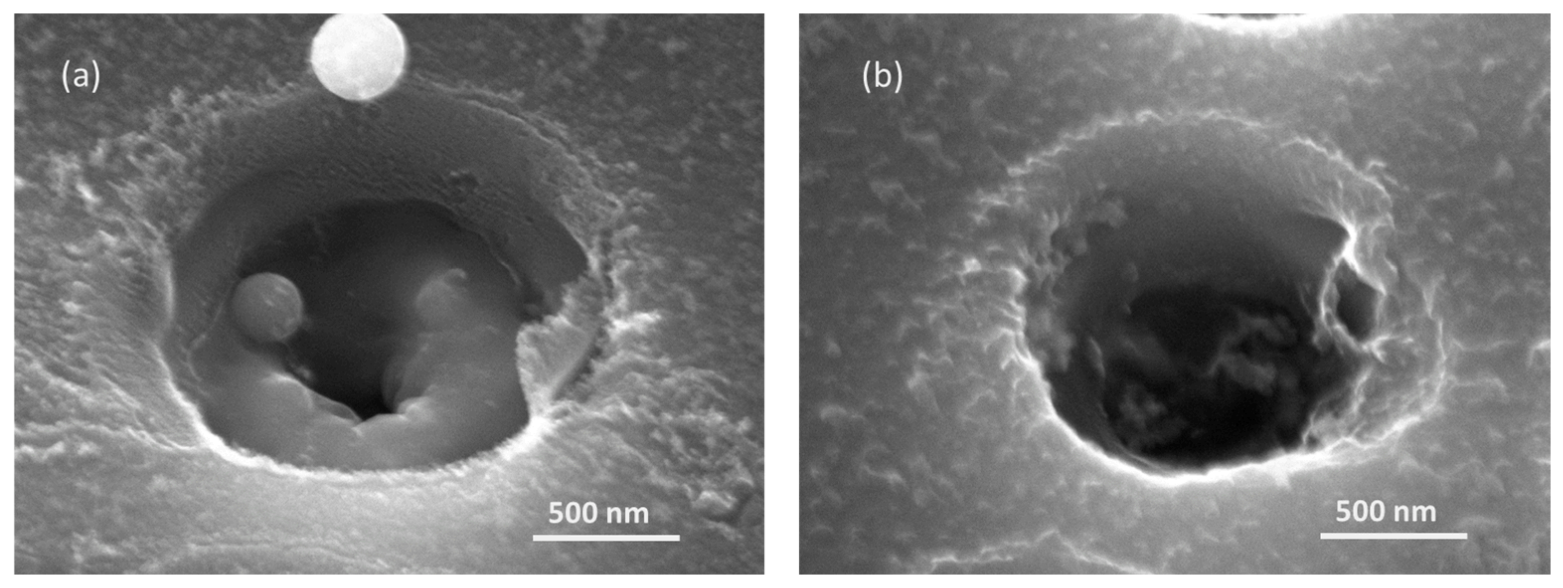
SEM images of a cavity with complete removal of the DLC layer: (**a**) before KOH wet etch; and (**b**) after the wet etch. The sample was tilted at 35° in the SEM chamber in (**a**,**b**).

The laser-patterned DLC on Si was submerged in the previously described KOH solution for a predefined amount of time (the etching time can be found in Supplementary Section S3). Then, the sample was taken out and rinsed in deionized water and dried in an oven at 75 °C for 40 min (according to S3, this etch time would yield at least 2 µm of Si removal). The sample was then inspected using SEM at a 35° tilt angle. The typical observation before and after the chemical etch is illustrated in Figure 4. The cavity appears to be rather deep, at least 2 µm in depth, and the aspect ratio of the cavity depth to diameter is larger than 1. Due to the limitation of the view, it is not possible to visualize the atomic plane formation at the bottom of the cavity. In order to achieve a more accurate evaluation of the geometry and the depth of the cavity, an FIB-based cross-sectional analysis is being planned.

One can visualize from Figure 4a a well-defined circular opening of the DLC and underlying Si. Due to DLC’s marked resistance to KOH compared to Si (0.04 nm/min for DLC vs. a few µm/min for Si), DLC serves as an excellent etch mask for the Si etching process [31]. After KOH etch, the exposed Si was completely etched away, revealing a hollow space underneath the DLC opening.

### 3.4. Dry Plasma Etch

**Figure 5:**
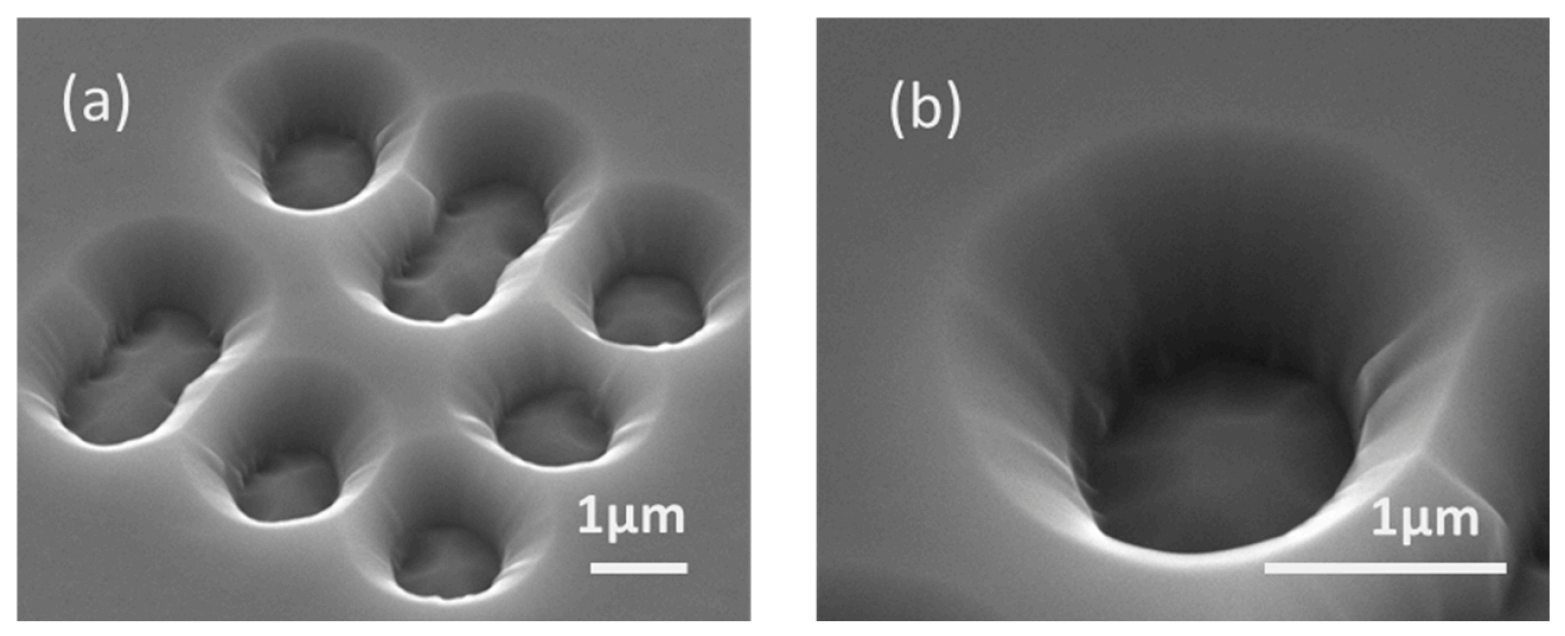
SEM images illustrating the matrix and individual cavity morphology after plasma etch: (**a**) the matrix of cavities; and (**b**) an individual cavity at high magnification. The sample was tilted at 35° in the SEM chamber in (**a**,**b**).

A typical plasma etching of the DLC on Si is shown in Figure 5. We observe from Figure 5 that a 3 × 3 cavity matrix sample at 2 µm pitch was chosen to undergo the plasma etching process. No additional cleaning of the sample was performed prior to and after the plasma treatment. The plasma etch was carried out in the abovementioned system at 0.7 Pa pressure and 20 °C ambient temperature, with a gas mixture of Cl_2_ (22 standard cubic centimeters per minute, sccm) and O_2_ (3 sccm). The chamber was first passivated, followed by an etching step of 800 s. Si etching in Cl_2_/O_2_ ICP-RIE plasmas is mainly driven by the formation of volatile silicon chlorides:

Si + 2Cl_2_ → SiCl_4_(g) (4)

Oxygen contributes to the formation of a thin oxidized surface layer, which competes with the chlorination process:

Si + O_2_ → SiO_x_ (5)

This oxidized layer is subsequently removed in the chlorine-based plasma, leading to volatile silicon-containing species, while oxygen is released in secondary reaction pathways. Ion bombardment enhances bond breaking and desorption of reaction products, resulting in anisotropic etching profiles. In addition, the O_2_ ambient could also etch DLC [32,33]. Nevertheless, preliminary results suggest the etch rate of DLC is less than 500 nm/400 s (Supplementary Section S3), slower than that of silicon. The exact etch rate of the DLC layer in the current condition is still under investigation.

The plasma-etched samples are then examined using SEM (tiled at a 35-degree angle similar to the previous section), as shown in Figure 5. It should be noted here that the imperfection in the location of the individual cavities was not due to the laser or plasma process but the positioning precision limit of the translation stage system. Apart from that, the sample surface is free of redeposition (Figure 5a)—the majority of the burr, dust, and particles generated during laser ablation were removed during the plasma treatment (see Figure 3 for comparison). Some curtain-like features can be seen on the sidewall of the cavities, which could have been formed during the plasma process. The diameter of the cavity at the entry is slightly enlarged. We could attribute this to the DLC etching in the plasma ambient that somehow compromised the anisotropic etching profile. The total depth of the cavity is measured to be approximately 1.6 µm after the plasma treatment. The depth-to-diameter ratio is close to but not necessarily surpass the factor 1. Nonetheless, this close-to-unity aspect ratio could still render the surface with the desired anti-reflection property, as is discussed in the following section.

### 3.5. Evaluation of the Anti-Reflection Performances

If the cavity structure is realized on an extended surface area (2-inch wafer, for instance), it can exhibit anti-reflective properties in the far infrared spectral region, making it suitable for applications in infrared optical systems. In this section, the reflectance and transmittance of the proposed structure are numerically simulated and analyzed.

#### 3.5.1. DLC Coated Si, Flat and Structured

**Figure 6:**
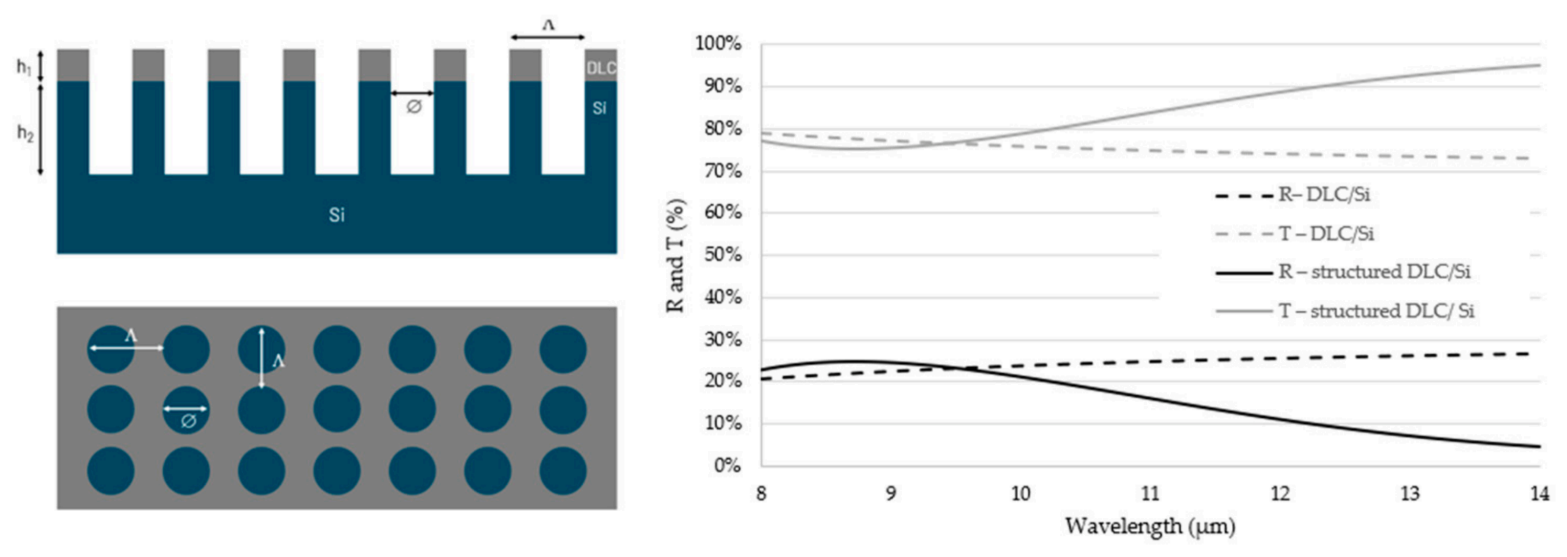
Simulated DLC-coated Si structures and corresponding optical response in the 8–14 µm infrared range. The structured surface was modeled as a periodic array of cylindrical cavities in silicon covered by a DLC layer, with pitch Λ = 2 µm, cavity diameter Ø = 1.6 µm, DLC coating thickness h_1_ = 0.4 µm, and cavity depth in Si h_2_ = 1.6 µm. Reflectance and transmittance spectra are compared to flat and structured DLC/Si surfaces.

Figure 6 shows the calculated reflectance and transmittance spectra of the flat DLC-coated silicon surface and of the structured DLC-coated silicon surface in the 8–14 µm infrared spectrum range. For the flat DLC-coated Si configuration, the reflectance remains relatively high over the whole spectral range, increasing from about 20% at 8 µm to approximately 27% at 14 µm. The corresponding transmittance slightly decreases from about 79% to 73%. After structuring, the optical response is significantly modified. The reflectance of the structured DLC-coated Si surface first remains close to that of the flat configuration in the shorter-wavelength part of the investigated range, then decreases markedly above approximately 9.5 µm, reaching values below 10% at longer wavelengths and about 5% at 14 µm. At the same time, the calculated transmittance increases continuously and reaches approximately 95% at 14 µm.

#### 3.5.2. Flat Si and Structured Si

**Figure 7:**
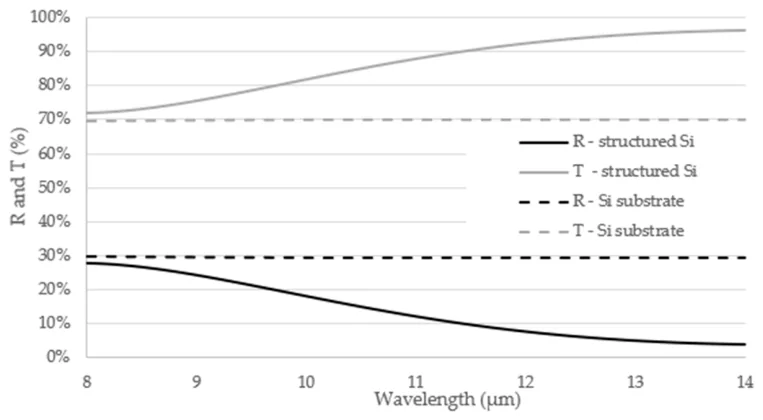
Optical response in the 8–14 µm infrared range. The structured surface was modeled as a periodic array of cylindrical cavities in silicon, with pitch Λ = 2 µm, cavity Ø = 1.6 µm, DLC layer thickness h_1_ = 0 µm, and cavity depth in silicon h_2_ = 1.6 µm. Reflectance and transmittance spectra are compared to flat Si substrates.

After plasma etching, the DLC layer is not preserved on the structured surface. The optical response was therefore also calculated for the corresponding structured silicon surface, in order to estimate the effect of the cavity array after removal of the DLC coating. As shown in Figure 7, the structured silicon surface exhibits a clear decrease in reflectance over the 8–14 µm range compared with the flat silicon substrate. The reflectance decreases from about 28% at 8 µm to below 5% at 14 µm, while the transmittance increases from approximately 72% to nearly 96%. This result indicates that the surface structuring itself provides an antireflective effect, particularly at longer wavelengths. The results of the performed simulations indicate that the fabricated surface structuring can improve the anti-reflective behavior of the DLC-coated silicon or silicon surface in the 9.5–14 µm range, with a particularly pronounced effect at the longer wavelengths. This improvement can be attributed to the structured morphology, which reduces the abrupt refractive-index transition at the air/DLC/silicon or air/silicon interface and promotes a more gradual effective optical response. It is worthwhile to mention that the present simulations were performed on the nominal geometry targeted by the fabrication process and experimentally obtained after structuring; they therefore provide an assessment of the anti-reflective behavior of the fabricated surfaces. Further parametric investigations, including the period, cavity diameter, depth, and DLC thickness, would be needed to optimize the geometry of the DLC/Si stack.

## 4. Discussion

As shown in Section 3.1, femtosecond laser processing using a Bessel beam configuration is well suited for the fabrication of densely packed micrometer-scale cavities on DLC-coated silicon. However, multiple pulse irradiation is required to achieve the desired cavity geometry. This sequential processing strategy is inherently slow because each cavity must be fabricated individually. Process parallelization through multiple Bessel beamlets could significantly reduce the overall fabrication time [34]. We have recently demonstrated beam shaping using a spatial light modulator (SLM, PLUTO-2.1-NIR-149, HOLOEYE Photonics AG, Berlin, Germany) to generate up to 20 simultaneous Bessel beamlets. Although this approach could theoretically reduce the processing time by approximately a factor of 20, the throughput remains insufficient for industrial implementation. Based on the simulation presented in Section 3.1, fabrication of a single wafer would still require more than 450 h even with 20 beamlets. More importantly, the conventional move-and-shoot strategy fundamentally limits scalability for large-area surface processing.

The burst-mode laser processing approach described in Section 3.2 provides a practical solution by enabling high-speed, on-the-fly fabrication, in which the sample moves continuously beneath a stationary laser beam. Each laser burst consists of a predefined number of pulses per burst (ppb). Because the sample translation speed is only on the order of several tens of millimeters per second, whereas the temporal separation between pulses within a burst is in the nanosecond range, the displacement of the sample during a burst is negligible. Consequently, all pulses within a burst interact with essentially the same location, collectively ablating the DLC layer to form a single cavity. In the present study, complete removal of the DLC coating required five consecutive bursts, each containing 25 ppb. This limitation originates primarily from the maximum ppb available from the laser source rather than from the process itself. Commercial laser systems capable of delivering a single burst containing 125 ppb, such as the Satzuma X system from Amplitude Systems (https://amplitude-laser.com/products/femtosecond-lasers/lasers-for-industry/satsuma-x/, accessed on 3 September 2026), are expected to achieve complete coating removal in a single burst. Preliminary experiments confirmed this capability, although these results are beyond the scope of the present work. Such laser systems would enable true on-the-fly fabrication by eliminating the need for repeated burst exposure at each processing location, thereby substantially increasing process throughput.

Following laser patterning, the exposed silicon beneath all openings is simultaneously removed by etching, making this step inherently parallel. Both investigated etching approaches exhibit distinct advantages and limitations. Potassium hydroxide (KOH)-based wet etching, investigated in Section 3.4, proved to be a viable fabrication route owing to the excellent chemical resistance of the DLC layer. Furthermore, multiple laser-patterned wafers can be processed simultaneously, making batch fabrication feasible. Nevertheless, wet etching exhibits several limitations related to etch uniformity and profile control. The resulting cavity geometry is strongly influenced by the anisotropic etch rates of different silicon crystallographic planes, while the final surface roughness also depends on crystal orientation [17]. A more detailed investigation of these effects will be the subject of future work. In the present study, Si (110) substrates were selected as a suitable compromise between etch profile and process performance. The rationale for this choice is provided in Supplementary Section S2.

Plasma etching provides an attractive alternative to KOH-based wet etching. As demonstrated in Section 3.4, plasma processing produces cleaner surfaces, eliminates the need for post-etch rinsing, and, for the etch depths investigated here, is essentially independent of silicon crystallographic orientation. Consequently, plasma etching can be applied to a broad range of silicon substrates rather than being restricted to Si (110), thereby improving process flexibility and scalability. Despite these advantages, plasma etching requires specialized equipment and chlorine-based process gases, resulting in higher processing costs. In addition, the plasma process may partially or completely remove the DLC layer. Although the optical performance is slightly improved after complete DLC removal, as shown in Figure 7 and discussed in Section 3.5, the DLC coating provides additional benefits including enhanced hardness, wear resistance, and environmental protection. To preserve these functionalities, post-etch re-deposition of the DLC coating represents a promising strategy. The feasibility of this approach is currently under technical evaluation.

It is worth noting that the simulations were performed using a simple geometry based on the nominal structural parameters targeted by the fabrication process. The experimental structures exhibit dimensions close to these nominal values, although deviations from the cylindrical geometry and periodic arrangement may arise from limitations in the positioning accuracy and from the laser and plasma processing steps. The simulations should therefore be considered as an assessment of the expected optical response of structures representative of the fabricated samples, rather than as an exact reproduction of their individual geometrical features. The actual optical performance of the fabricated structures must ultimately be measured and evaluated experimentally. As a near-future perspective, the proposed hybrid laser-patterning and etching process will be extended to fabricate functional surface structures on 2-inch DLC-coated silicon wafers. The fabricated samples will be characterized to evaluate their antireflective performance in the infrared spectral range. In addition to improving optical performance, the hybrid surface texturing process may also modify the wettability of the DLC-coated silicon surface [35]. Enhanced hydrophobicity could provide additional functionalities, including anti-fogging and self-cleaning behavior. Upon successful validation, these structured surfaces are envisioned for use as optical windows in night-vision, surveillance, and infrared imaging systems. Although DLC-coated silicon serves as the primary demonstration platform in this study, the proposed methodology is readily transferable to other substrate materials. In particular, DLC-coated germanium is a promising candidate because of its excellent infrared transmission and superior antireflective performance in the far-infrared spectral region.

## 5. Conclusions

A hybrid laser–etching process was developed for the fabrication of high-density, micrometer-scale deep cavities on DLC-coated silicon substrates. By combining on-the-fly burst-mode laser ablation with a subsequent etching step, the proposed process offers high scalability and significant potential for industrial manufacturing. A Bessel beam was employed to generate a tightly focused laser beam with an extended non-diffractive propagation length, while burst-mode irradiation selectively removed the DLC coating from Si (110), producing well-defined openings for subsequent silicon etching. The etching process then selectively removed silicon beneath these openings, enabling the fabrication of high-aspect-ratio microstructures. Simulations confirm that the resulting surface textures should exhibit excellent antireflective properties, demonstrating strong potential for optical window applications in night-vision and surveillance systems.

## Data Availability

The original contributions presented in this study are included in the article and Supplementary Material. Further inquiries can be directed to the corresponding authors.

## Supplementary Materials

The following supporting information can be downloaded at: https://www.mdpi.com/article/10.3390/nano16181131/s1, Figure S1.1: A SEM micrograph illustrating a matrix of laser impacts made with a Gaussian beam for ablation threshold evaluation and for KOH etch rate evaluation; Figure S2.1: SEM images illustrating KOH etching geometries: (a) DLC on Si (110), the etching terminates with 2 inclined (111) crystalline faces underneath the DLC opening; and (b) DLC on Si (111), the etching terminates with 4 inclined (111) crystalline faces forming an inversed pyramid. It is also noted that the etching profile is enlarged to a square base underneath the circular DLC opening; Figure S3.1: Confocal microscope re-constructed 3D surface profiles of laser impact before and after the wet KOH etch; Figure S3.2: Confocal microscope re-constructed 3D surface profile of a laser impact less deep that the DLC layer, before (left) and after the plasma etch (right); Figure S3.3: Confocal microscope re-constructed 3D surface profiles of laser impact after the plasma etch, 400 s; Figure S4.1: Optic microscope images showing sample surface patterned in on-the-fly configuration; Figure S5.1: Beam analysis of the Bessel beam; Table S1.1: the ablation threshold values as function of number of shots; Table S1.2: the ablation depth as function of shots and bursts. Laser repetition rate was fixed at 50 kHz. Note: the italic fonts indicate the DLC layer was pierced through; Table S2.1: Etch rate of Si (100) and Si (110) in KOH; Table S2.2: Etch rate of Si (100) coated with 1.4 µm thick DLC.

## Abbreviations

The following abbreviations are used in this manuscript:

fs	femtosecond
ps	picosecond
ns	nanosecond
ms	millisecond
µm	micrometer
ppb	Pulse per burst
kHz	Kilo Hertz
GHz	Giga Hertz
DLC	Diamond-like carbon
Si	Silicon
KOH	Potassium hydroxide solution
sccm	Standard Cubic Centimeter per Minute
