# Supplementary material for: Hybrid Laser–Etching Fabrication of High-Density Antireflective Microstructures on DLC-Coated Silicon Using Burst-Mode Bessel Beam Processing
Source: Nanomaterials (Basel). 2026 Sep 10;16(18):1131. doi: 10.3390/nano16181131 (PMC13610680; doi:10.3390/nano16181131)
Supplement: Supplementary file 1 [file nanomaterials-16-01131-s001.zip › nanomaterials-4497493-supplementary.pdf]

Communication

Hybrid Laser–Etching Fabrication of High-Density Antireflective Microstructures on DLC-Coated Silicon Using Burst-Mode Bessel Beam Processing

Supplementary Materials:

The Supporting Information is organized as follows. Section S1 presents the investigation of the ablation threshold and material removal under both conventional single-pulse and burst-mode irradiation. Section S2 describes the rationale for selecting the crystallographic orientation of the silicon substrate for KOH etching. Section S3 provides an estimation of the etching time. Next, Section S4 investigates the optomechanical aspects of the on-the-fly processing configuration. Finally, Section 5 describes the Bessel beam characteristics. The investigations reported in Sections S1–S3 were performed using a conventional Gaussian laser beam with a diameter of approximately 30 μm, rather than the Bessel beam configuration used in the main experiments. The Gaussian beam was selected to facilitate the characterization and quantitative analysis of the laser-patterning and etching results at lateral dimensions of several tens of micrometers, rather than at sub-micrometer dimensions. In particular, the larger features enabled more reliable measurements of crater diameter and depth before and after etching.

The authors acknowledge that Gaussian and Bessel beams exhibit fundamentally different spatial energy distributions, which can lead to different energy-deposition conditions, thermal diffusion dynamics, and plasma-shielding effects during laser–matter interaction. Therefore, in Section S1, quantitative differences may exist between the ablation efficiency measured using the Gaussian beam and that obtained under Bessel-beam irradiation. Similarly, in the geometrical characterization and etching-time estimation presented in Section S3, some differences may arise between structures produced with Gaussian and Bessel beams. In particular, the different dimensions and geometries of the resulting cavities may affect the wetting behavior and, consequently, the etching process. Nevertheless, these experiments provide a reasonable first-order estimation of the relevant processing parameters and trends. Their purpose is to establish a simplified and experimentally accessible basis for characterizing the laser ablation and subsequent etching processes, rather than to provide a direct quantitative reproduction of the Bessel-beam processing conditions. In contrast, Section S4 specifically investigates the optomechanical performance of the on-the-fly processing configuration. A Bessel beam was therefore used in this section, consistent with the main experimental configuration. The laser triggering and continuous sample motion were synchronized to demonstrate that the positioning precision achieved during on-the-fly processing is sufficient for the fabrication of regularly patterned structures over large surface areas. As supporting information, Section S5 details the geometry feature of the Bessel beam used in this study.

S1. Burst efficiency compared with classic single-shot irradiation efficiency

As explained above, the systematic investigation of the ablation threshold and ablation depth was performed using a conventional Gaussian beam rather than the Bessel beam configuration. A conventional Gaussian beam was used for the systematic investigation

of the ablation threshold and material removal instead of the Bessel beam configuration. This choice was made to simplify the characterization and quantitative analysis of the laser-induced craters. Unlike Bessel beams, which exhibit an extended focal region and a more complex transverse intensity distribution, Gaussian beams produce well-defined and approximately circular ablation features. Their diameter can therefore be more readily measured by optical microscopy, while their depth and morphology can be accurately characterized by confocal microscopy. The use of a Gaussian beam thus provides a simpler and more reliable basis for determining the ablation threshold and quantifying material removal as a function of laser pulse energy and number of pulses. In this study, a ZEISS optical microscope (Oberkochen, Germany) was used to measure the crater diameters, while the topography and depth of the laser-affected craters were characterized using a chromatic confocal microscope (STIL, Altimet, France). A representative matrix of laser impacts obtained for different laser pulse energies and numbers of laser shots is shown in Fig. S1.1.

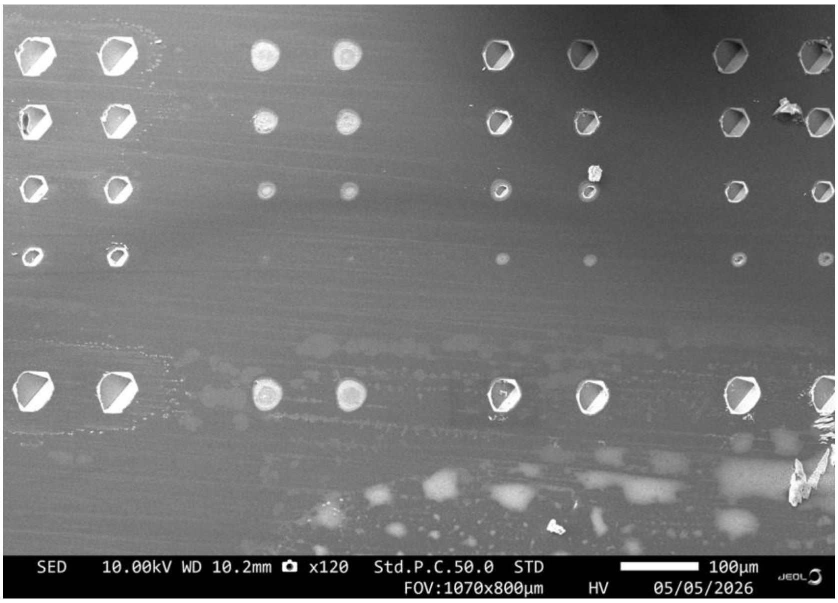

Figure S1.1. An SEM micrograph illustrating a matrix of laser impacts made with a Gaussian beam for ablation threshold evaluation and for KOH etch rate evaluation.

The laser ablation thresholds were subsequently determined using the D^2 method, and the resulting values are summarized in Table S1.1. For both conventional kHz repetition-rate irradiation and burst-mode processing with 5 and 25 pulses per burst (ppb), a decrease in the ablation threshold is observed as the number of laser shots or bursts increases. In burst mode, the rapid succession of pulses can reduce the effective ablation threshold because subsequent pulses interact with a pre-heated and thermally confined surface layer before significant heat dissipation occurs. This cumulative thermal effect reduces the energy required for material removal and may explain the general trend observed in Table S1.1. In particular, the ablation threshold values obtained with 5 pulses per burst (ppb) are lower than those measured under conventional kHz-repetition-rate irradiation with multiple pulses, as shown in the first and second columns of Table S1.1. However, the threshold values increase again when 25 ppb is used (last column). This increase can be attributed to the significantly lower energy delivered per pulse within the 25-ppb burst. The energy deposited by each individual pulse may then be insufficient to efficiently pre-heat the material toward the thermal conditions required for ablation, thereby reducing the cumulative thermal enhancement (https://doi.org/10.1364/OE.400624). The lowest ablation threshold in the present set of

experiments was observed for 125 bursts in the 5-ppb configuration. This threshold was approximately seven times lower than those obtained under neighboring irradiation conditions, such as 125 pulses at the conventional kHz repetition rate and 25 bursts in the 5-ppb configuration. This pronounced reduction may result from the combined effects of GHz burst-induced pre-heating and the incubation effect during DLC ablation. A reduction in the ablation threshold by approximately one order of magnitude between single-shot and multiple-shot irradiation has previously been reported for DLC and was attributed to the gradual accumulation of laser-induced defects in the material (https://doi.org/10.1016/j.optmat.2022.112203). The simultaneous contribution of incubation over successive bursts and cumulative thermal effects within each GHz burst may therefore explain the approximately seven-fold reduction in the threshold observed for 125 bursts at 5 ppb.

Table S1.1: The ablation threshold values as a function of the number of shots

	$F_{th\_classic}$	F_{th_5ppb}	F_{th_25ppb}
1 shot	1.62 J/cm^2	1.55 J/cm^2	1.76 J/cm^2
5 shots/bursts	1.03 J/cm^2	0.48 J/cm^2	1.1 J/cm^2
25 shots/bursts	0.92 J/cm^2	0.42 J/cm^2	0.88 J/cm^2
125 shots/bursts	0.4 J/cm^2	0.06 J/cm^2	0.7 J/cm^2

Furthermore, the ablation depths in the DLC coating layer is summarized in Table S1.2 below (the thickness of the DLC is 1.4 µm in this study). The table shows that at a fixed laser power setting, burst modes were always more efficient in terms of material removal, with the only exception at the lowest laser power of 80 mW, where the burst modes showed less material removal. It should be noted that for crater depth more than 1.4 µm means the ablation reached Si substrate. These values are written in *italic* in the table.

Table S1.2: The ablation depth as a function of shots and bursts. Laser repetition rate was fixed at 50 kHz. Note: the italic fonts indicate the DLC layer was pierced through.

	Classic, 1 pulse	5 ppb, 1 burst	25 ppb, 1 burst
80 mW	0.4 µm	0.25 µm	0.36 µm
160 mW	0.6 µm	1.4 µm	*1.8 µm*
320 mW	0.7 µm	*1.9 µm*	*1.9µm*
640 mW	0.74 µm	*2.52 µm*	*1.9 µm*
1.2 W	0.7 µm	*2.58 µm*	*2.3 µm*

S2. The choice of the crystal orientation of the silicon substrate

The finishing geometry of the wet etch strongly depends on the crystalline orientation. Chemical etching of Si in KOH solution comprises two main steps: the OH−catalyzed conversion of Si–H surface bonds to give Si–OH (and H_2) and the breaking of the polarized Si–Si back-bond by reaction with water. The latter step regenerates the surface hydride. The atomic arrangement depends on the surface: (100), (110), and (111) surface atoms have 2, 2, and 3 Si–Si back-bonds, respectively. Therefore, for the (111) case, the surface atoms are more firmly anchored in the lattice. It is more difficult to form the transition-state

intermediate and the surface is more stable. Typical etch profiles of a Si (110) and a Si (100) under the DLC opening are shown in Figure S2.1.

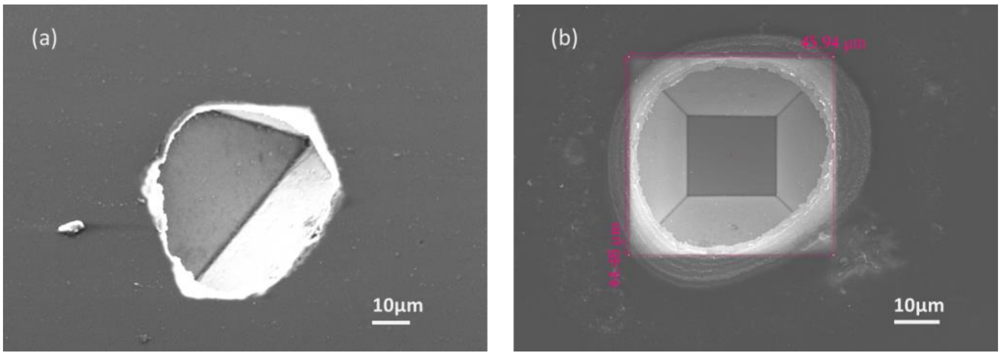

Figure S2.1. SEM images illustrating KOH etching geometries: (**a**) DLC on Si (110), the etching terminates with 2 inclined (111) crystalline faces underneath the DLC opening; and (b) DLC on Si (111), the etching terminates with 4 inclined (111) crystalline faces forming an inverted pyramid. It is also noted that the etching profile is enlarged to a square base underneath the circular DLC opening.

It is observed in Fig. S2.1b that the KOH etch removed Si not only underneath the circular opening of the DLC layer, but also spread out to form an inverse pyramid profile, an undercut, due to the preferential etch stops of the 4 (111) planes.

S3. Etching rate estimation

Wet etch in KOH

The etching rate was also investigated using a classic Gaussian beam instead of a Bessel beam configuration for the same reason as stated above. A chromatic confocal microscope (Stil, of Altimet, France) was used to evaluate the depth evolution before and after the wet etch in KOH, as well as the plasma etch in Cl_2 and O_2.

Figure 3.1 illustrates the 3D profiles of a laser-irradiated DLC on Si (100) sample before and after the KOH etch. The sample was submerged in 35wt% KOH bath at 85 °C for 5 minutes. It is evident the laser ablated craters became deeper after the KOH bath due to the etch effect.

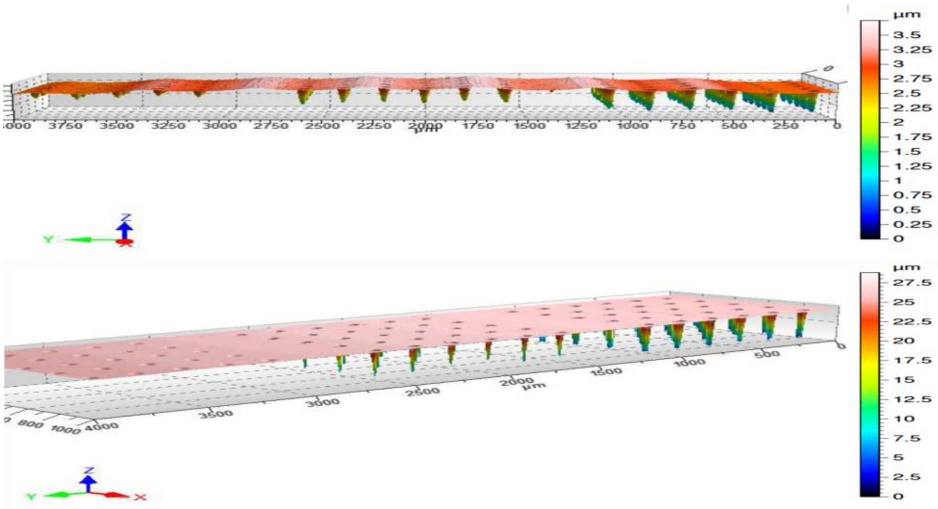

Figure S3.1. Confocal microscope re-constructed 3D surface profiles of laser impact before and after the wet KOH etch.

It was found out that the etch reproducibility is better when the bath temperature was regulated at 75 °C. The etch rate was deduced for both DLC on Si (100) and DLC on Si

(110). The etch rates are listed in Table S2.1. The etch rate is between 0.46 and 0.61 μm/minute for Si (100) and 0.05 to 0.06 μm/minute for Si (110).

Table S2.1: Etch rate of Si (100) and Si (110) in KOH

	5 minutes	10 minutes	20 minutes
Si (100)	2.77 μm	4.59 μm	12.29 μm
Si (110)	0.27 μm	0.45 μm	1.3 μm

Plasma dry etch

Some plasma-etched DLC on Si samples are shown in Figure S3.2 and Figure S3.3. The plasma etch rate estimation is in a similar way as for KOH. Table 3.2 summarizes the etch depths of different cavities.

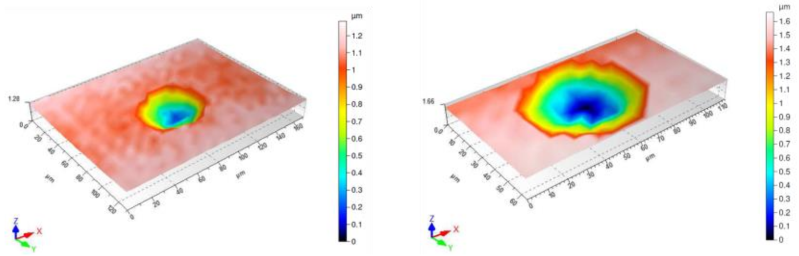

Figure S3.2. Confocal microscope re-constructed 3D surface profile of a laser impact less deep than the DLC layer, before (left) and after the plasma etch (right).

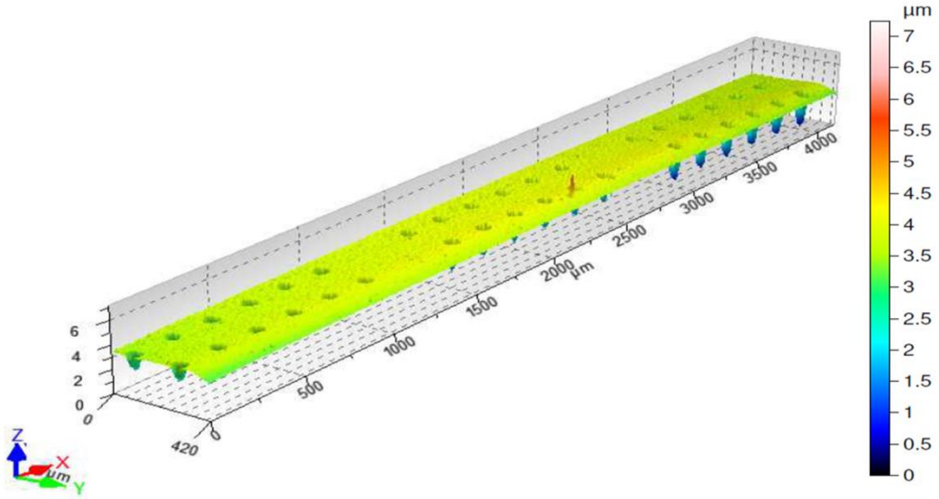

Figure S3.3. Confocal microscope re-constructed 3D surface profiles of laser impact after the plasma etch, 400 seconds.

Since the initial DLC layer thickness was 1.4 μm, the dry plasma etch rate of DLC can be estimated to be approximately 0.5 μm per 400 s, as shown in the second column of Table S2.2. For the samples in which laser irradiation completely penetrated the DLC layer, the corresponding cavity depths are indicated in italics. A larger variation in cavity depth can be observed for these samples, which can be attributed to the higher plasma etch rate of silicon compared with DLC. Based on the values reported in the table, the silicon etch rate can be estimated to be approximately 0.9–1.1 μm per 400 s. The plasma etch rate of silicon is twice as fast as that of the silicon.

Table S2.2: Etch rate of Si (100) coated with 1.4 μm thick DLC.

Initial depth	0.9 μm	*1.6 μm*	*2.2 μm*
Etch depth 400 sec	1.4 μm	*2.5 μm*	*3.4 μm*

From this investigation, it is concluded that the plasma dry etch of silicon is roughly twice faster than that of the DLC coating.

S4. Surface patterning in on-the-fly configuration

In this section, laser irradiation was performed in an on-the-fly configuration, with the sample mounted on a linear translation stage moving continuously without stopping. A 0.2 × 0.2 mm^2 matrix was fabricated with a pitch of 2 μm using a Bessel beam on a silicon substrate. For the sake of simplicity, and to demonstrate that sufficient positioning precision could be achieved during continuous motion, single-pulse irradiation was used. Figure S4.1 shows optical microscope images of the fabricated matrix at different magnifications.

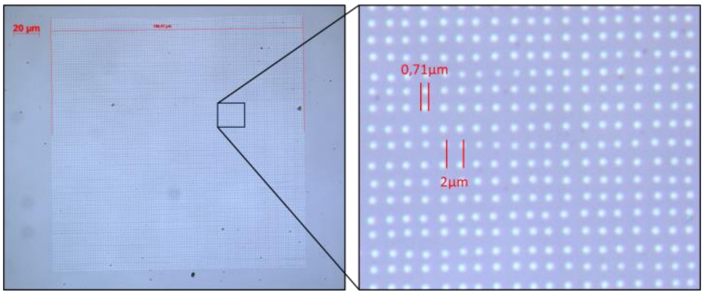

Figure S4.1. Optical microscope images showing sample surface patterned in on-the-fly configuration.

The laser pulse energy was set slightly below the ablation threshold. Under these conditions, a clear optical contrast was observed, which is attributed to the refractive-index modification associated with femtosecond laser-induced amorphization of silicon. The higher-magnification image on the right clearly reveals the positioning accuracy through the regular array of laser-induced amorphized spots. This preliminary demonstration confirms the capability of the on-the-fly processing configuration to pattern large surface areas with a regular pitch. Nevertheless, the positioning accuracy achieved experimentally is not as ideal as that assumed in the numerical simulations. Therefore, the actual optical performance of the laser-patterned samples must ultimately be evaluated experimentally.

S5. Bessel beam characteristics

As shown in the report, the axicon apex angle is 176°, the base angle is α = 2°, and the refractive index is n = 1.46 (taken from the supplier Thorlabs' datasheet). The cone angle is:

$\theta = \arcsin(n \sin\alpha) - \alpha \approx 0.92° \approx 0.0161\ rad.$

For an ideal zero-order Bessel beam with the intensity profile

$I(r) = I_0 J_0^2(k_r r),$

the FWHM diameter of the central lobe is approximately:

$D_{FWHM} \approx \sin\theta \times 0.359\lambda$, therefore

$D_{FWHM} \approx \sin(0.92°) \times 0.359 \times 1.03 \approx 23\ \mu m.$

After the 4f system (200 mm and 10 mm), the Bessel beam size (central core, FWHM) is 1.15 μm.

For an ideal zero-order Bessel beam, the first side-lobe maximum occurs at approximately:

$k_r r \approx 3.832$

At this position:

$I_{1st\text{-}lobe}/I_{core} \approx 0.162$

Therefore, the energy contrast is 16% in theory. Experimentally, the intensity of the first side lobe relative to the peak intensity of the central core was estimated to be below 25% (beam analysis measurements; the beam analyzer consisted of a very similar objective lens and a tube lens, and a Thorlabs camera).

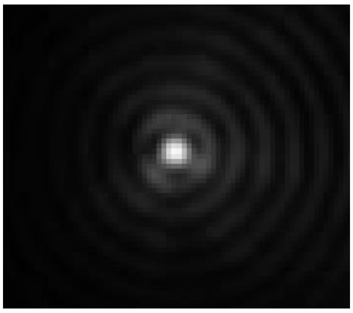

Figure S5.1. Beam analysis of the Bessel beam
